# Immunoreactivity of Vesicular Glutamate Transporter 2 Corresponds to Cytochrome Oxidase-Rich Subcompartments in the Visual Cortex of Squirrel Monkeys

**DOI:** 10.3389/fnana.2021.629473

**Published:** 2021-02-18

**Authors:** Songping Yao, Qiuying Zhou, Shuiyu Li, Toru Takahata

**Affiliations:** ^1^Key Laboratory for Biomedical Engineering of Ministry of Education, College of Biomedical Engineering and Instrument Science, Zhejiang University, Hangzhou, China; ^2^Interdisciplinary Institute of Neuroscience and Technology, Zhejiang University School of Medicine, Hangzhou, China; ^3^Department of Neurology of the Second Affiliated Hospital, Zhejiang University School of Medicine, Hangzhou, China

**Keywords:** Slc17a6, *Saimiri sciureus*, MAB5504, CO blob/puff/patch, parallel visual pathways, New World monkeys

## Abstract

Cytochrome oxidase (CO) histochemistry has been used to reveal the cytoarchitecture of the primate brain, including blobs/puffs/patches in the striate cortex (V1), and thick, thin and pale stripes in the middle layer of the secondary visual cortex (V2). It has been suggested that CO activity is coupled with the spiking activity of neurons, implying that neurons in these CO-rich subcompartments are more active than surrounding regions. However, we have discussed possibility that CO histochemistry represents the distribution of thalamo-cortical afferent terminals that generally use vesicular glutamate transporter 2 (VGLUT2) as their main glutamate transporter, and not the activity of cortical neurons. In this study, we systematically compared the labeling patterns observed between CO histochemistry and immunohistochemistry (IHC) for VGLUT2 from the system to microarchitecture levels in the visual cortex of squirrel monkeys. The two staining patterns bore striking similarities at all levels of the visual cortex, including the honeycomb structure of V1 layer 3Bβ (Brodmann's layer 4A), the patchy architecture in the deep layers of V1, the superficial blobs of V1, and the V2 stripes. The microarchitecture was more evident in VGLUT2 IHC, as expected. VGLUT2 protein expression that produced specific IHC labeling is thought to originate from the thalamus since the lateral geniculate nucleus (LGN) and the pulvinar complex both show high expression levels of *VGLUT2* mRNA, but cortical neurons do not. These observations support our theory that the subcompartments revealed by CO histochemistry represent the distribution of thalamo-cortical afferent terminals in the primate visual cortex.

## Introduction

The primate visual system is subdivided into multiple parallel pathways, and this segregation appears as laminar and columnar domains in the visual cortex (Sincich and Horton, 2005). Some of these domains have been revealed using cytochrome oxidase (CO) histochemistry, such as blobs/puffs/patches in superficial layers of V1 as well as thick, thin, and pale stripes in the middle layer of V2 (Horton and Hubel, 1981; Horton and Hocking, 1996). CO is the most common metabolic enzyme found in mitochondria, and its enzymatic activity is coupled with neuronal activity, which is indicated by decreased CO expression in ocular dominance columns (ODCs) that occurs after monocular inactivation (Horton and Hedley-Whyte, 1984; Wong-Riley, 1989). Biochemical assays also demonstrated that mitochondrial transcription of metabolic genes is dependent on neuronal activity (Wong-Riley, 2012). Therefore, previous researchers thought that neurons in CO-rich domains are more active than those outside the domains.

However, we questioned this paradigm (Takahata, 2016), since neurons in the V1 blobs and V2 stripes do not show higher expression of immediate-early genes (IEGs), which is more directly coupled with neuronal spiking activity (Kim et al., 2010), compared to neurons outside V1 blobs or V2 dark stripes. Instead, the CO labeling pattern resembles the staining pattern observed for immunoreactivity (ir) of vesicular glutamate transporter 2 (VGLUT2). *VGLUT2* mRNA is abundantly expressed in sensory thalamic nuclei, and the protein product of *VGLUT2* is transported into axon terminals to support glutamate transmission at synapses, and its mRNA is not strongly expressed in cortical neurons (Nakamura et al., 2007; Balaram et al., 2013). VGLUT2-ir corresponded to anterogradely labeled thalamo-cortical axons, but not to cortico-cortical axons, in the ferret visual cortex (Nahmani and Erisir, 2005). Therefore, immunohistochemistry (IHC) for VGLUT2 is thought to reveal the distribution of thalamo-cortical afferent terminals in the cortex (Hackett and de la Mothe, 2009). Not only are the two laminar patterns similar, a corresponding pattern between CO blobs and VGLUT2-ir expression has been suggested in galagos, macaques, and humans (Garcia-Marin et al., 2013; Rockoff et al., 2014). In addition, the honeycomb structure of layer 3Bβ (Brodmann's layer 4A) is comparable between CO and VGLUT2 IHC (Garcia-Marin et al., 2013). No published report describes a V2 stripe-like staining pattern for VGLUT2-ir, however, traditional tracer studies have revealed that afferents from the pulvinar preferentially terminate into the thick and thin stripes of V2, but not the interstripe regions (Livingstone and Hubel, 1982). We consider that the thalamo-cortical afferent terminals may possess higher metabolic activity than the soma or axons of cortical neurons in general, and CO histochemistry reflects the distribution of thalamo-cortical axons and afferent terminals, rather than the spiking activity of cortical neurons when the cortex is stained (Takahata, 2016).

In this study, we compared the staining patterns between CO histochemistry and VGLUT2 IHC in tangential sections of the visual cortices of squirrel monkeys, including the blobs and honeycomb structures in V1 and stripes in V2. The squirrel monkey has not been used to study correspondence between CO subcompartments and VGLUT2-ir, but proved to be an excellent animal model because they have distinct V1 blobs and V2 stripes that are easily discernible in the tangential plane (Horton and Hocking, 1996). We demonstrate striking similar staining patterns between CO histochemistry and VGLUT2-ir from the system to the microstructure levels.

## Materials and Methods

The brains of three male and three female adult squirrel monkeys (*Saimiri sciureus*, body weight range of 900 to 1,100 g for males and 500 to 800 g for females) were used in this study. Among them, four hemispheres from three monkeys were used for cortical tangential staining: The right cortex of ID 18–20, male, 1,100 g, that was subjected to intravitreous injection of tetrodotoxin (TTX, 1 mM−8 μL) into the right eye 1 day before perfusion, the right and left cortices of ID 18–27, female, 500 g, that was subjected to intravitreous injection of TTX (1 mM−5 μL) into the left eye 2 days before perfusion, and the right cortex of ID 18–30, male, 1,000 g, that was subjected to left eye enucleation 14 days before perfusion. All procedures were approved by the Institutional Animal Care and Use Committee of Zhejiang University, and were in accordance with the guidelines of the National Institutes of Health (NIH), United States of America.

### Tissue Preparation

All animals were subjected to monocular inactivation treatment by either eye enucleation or TTX injection for 1 day to 3 weeks for a separate study. This treatment may have altered expression patterns of proteins, but at least, plastic changes of visual system organization were not expected since none of them received monocular inactivation when they were juvenile. Besides, the staining patterns for CO histochemistry were nearly identical to those described in previous reports for visually intact squirrel monkeys (Carroll and Wong-Riley, 1984; Wong-Riley and Carroll, 1984b). Patchy or stripe-like domains related to ocular dominance were not clearly observed in V1 in our staining, most likely because our subjects did not possess ocular dominance-segregation for the geniculo-cortical inputs (Adams and Horton, 2003).

The animals were given an overdose of sodium pentobarbital (>50 mg/kg body weight) and were perfused with a sucrose solution [8.5% sucrose, 5 mM MgCl_2_ in 20 mM phosphate buffer (PB)], followed by 0.5–4.0% paraformaldehyde (PFA) in 0.1M PB. The brain was removed from the skull, and the visual cortex was separated from the rest of the brain and flattened immediately. The flattened visual cortices were immersed in a post-fixative (30% sucrose/4% PFA in PB) at 4°C overnight and cut tangentially at 40 μm using a freezing microtome. The remaining brain tissue was immersed in 30% sucrose in PB at 4°C for ~1 week until the tissue sank to the bottom of the container. Then, the brain tissue was cut coronally at 40 μm using a freezing microtome. Sections were stored at −20°C in a cryoprotectant solution [30% ethylene glycol, 30% glycerol, and 40% phosphate-buffered saline (PBS)] until used.

### CO Histochemistry

CO histochemistry was conducted as described previously (Wong-Riley, 1979) with slight modifications. The free-floating sections were washed twice in 5% sucrose in PBS for 5–10 min. The sections were incubated in the CO reaction solution [200 μg/mL cytochrome C (Sigma-Aldrich, St. Louis, MO), 150 μg/mL catalase (Sigma-Aldrich), and 100 μg/mL 3,3′-diaminobenzidine (DAB; Sigma-Aldrich) in 5% sucrose and PBS] at 37°C, 30 rotation per minute (rpm) for 8–24 h. Subsequently, the sections were washed three times with PBS, mounted on glass microscope slides and air-dried. The sections on slide were dehydrated through a series of increasing ethanol concentrations followed by xylene, and permanently coverslipped with xylene-based glue. The dehydration/coverslipping procedure was the same in following other staining as well.

### Immunohistochemistry (IHC)

Commercially available antibodies were used in this study (summarized in Table 1). Mouse monoclonal anti-VGLUT2 was generated against a recombinant antigen. In Western blots of the primate neocortex, the antibody recognized a band at 56-kDa, the known molecular weight of VGLUT2 (Balaram et al., 2013; Baldwin et al., 2013). This monoclonal antibody has been used previously to label VGLUT2 in rodents (Wong and Kaas, 2008; Dondzillo et al., 2010), tree shrews (Balaram et al., 2015), sea lions (Sawyer et al., 2016), as well as primates (Balaram et al., 2013; Takahata et al., 2018).

**Table 1:** Information about antibodies used in this study.

**Antibody name**	**Host animal**	**Producing company**	**Catalog number**	**RRID**	**Supplied concentration**	**Dilution ratio to be used**
Anti-VGLUT2 monoclonal antibody	Mouse	Millipore, Bedford, MA	MAB5504	AB_2187552	1.0 mg/mL	1:30,000 for visual cortex
						1:10,000 for LGN and pulvinar
Anti-calbindin D-28K monoclonal antibody	Mouse	Sigma-Aldrich, St. Louis, MO	C9848	AB_476894	15–55 mg/mL	1:1,000
Anti-mouse IgG polyclonal antibody, biotinylated	Horse	Vector Laboratories, Burlingame, CA	BA-2000	AB_2687893	1.5 mg/mL	1:1,000

Free-floating sections were washed three times in 0.3% Triton X-100/PBS (PBST). Endogenous peroxide activity was quenched with 0.3% H_2_O_2_ in PBST for 15 min. Then, the sections were transferred into citrate buffer (0.3% Triton X-100 in 10 mM sodium citrate, pH 6.0), washed several times and incubated in citrate buffer at 80°C for 30 min for antigen retrieval. The sections were allowed to cool to room temperature (RT) and placed in blocking buffer (Roche Diagnostics, Indianapolis, IN) for 1 h, followed by incubating the sections overnight in primary antibody, anti-VGLUT2 or anti-calbindin D-28K (CB) (Table 1), at 4°C. After washing in PBST, the sections were incubated in secondary antibody (Table 1) for 2 h at RT. Then the sections were washed in PBST and transferred into a solution from VECTASTAIN® Elite ABC Kit (Vector Laboratories, Burlingame, CA) for 1 h according to the manufacturer's instructions. The sections were washed three times in PBST and placed in the reaction buffer (200 μg/mL DAB, 0.02% nickel chloride, 0.03% H_2_O_2_ in PBST) to visualize the signals. The sections were then washed with PBST three times, mounted on glass microscope slides and air-dried.

### *In situ* Hybridization (ISH)

To prepare the squirrel monkey *VGLUT2*-specific probe, a part of the *VGLUT2* gene was cloned using RT-PCR with cDNA prepared from an enucleated eye from one of the squirrel monkeys. The primer sequences were GGCAAGGTCATCAAGGAGAA (forward) and GCACAAGAATGCCAGCTAAAG (reverse) that targeted the 322–713 region of NM_020346 (human *VGLUT2/SLC17A6*). The PCR amplicon was purified and inserted into a plasmid vector, pCR™II-TOPO® vector, Dual Promoter (Invitrogen, Waltham, MA) using conventional TA cloning, and amplified in competent cells. The plasmids were harvested and purified with QIAGEN® Plasmid Midi Kit (Qiagen, Hilden, Germany) according to the manufacturer's instructions. For colorimetric ISH, digoxigenin (DIG)-labeled antisense and sense riboprobes were prepared from the plasmids using a DIG-dUTP labeling kit (Roche Diagnostics).

Our ISH protocol was based on previous works (Takahata et al., 2014) with slight modifications. Briefly, free-floating brain sections were immersed in 4% PFA in 0.1 M PB (pH 7.4) overnight at 4°C, then treated with 1–10 μg/mL proteinase K for 30 min at 37°C. After acetylation, the sections were incubated in the hybridization buffer [5x standard saline citrate (SSC: 150 mM NaCl, 15 mM sodium citrate, pH 7.0), 50% formamide, 2% blocking reagent (Roche Diagnostics), 0.1% N-lauroylsarcosine (NLS), 0.1% sodium dodecyl sulfate (SDS), 20 mM maleic acid buffer; pH 7.5] containing 1.0 μg/mL DIG-labeled riboprobe overnight at 60°C. Hybridized sections were washed twice, 20 min each in wash buffer (2x SSC, 50% formamide, 0.1% NLS) at 60°C. Subsequently, the sections were successively immersed in RNase A buffer [10 mM Tris-HCl, 10 mM ethylenediamine-N, N, N′, N′-tetraacetic acid (EDTA), 500 mM NaCl, pH 8.0] that contained 20 μg/mL RNase A for 30 min at 37°C, 2x SSC/0.1% NLS for 20 min at 37°C, and 0.2x SSC/0.1% NLS for 15 min at 37°C. Hybridization signals were visualized using alkaline phosphatase (AP) immunohistochemical staining and a DIG detection kit (Roche Diagnostics) that used an overnight reaction to nitro blue tetrazolium chloride/5-bromo-4-chloro-3-indolyl phosphate, toluidine salt (NBT/BCIP) (Roche Diagnostics). The sections were then washed with deionized water, mounted on glass microscope slides and air-dried.

### Nissl Staining

Free-floating sections were post-fixed in 4% PFA for a minimum of 12 h at 4°C. The sections were mounted on glass microscope slides after washing in 0.1 M PB and air-dried for several days. The slides with sections were then rinsed successively in deionized water, then 90 and 75% ethanol. The sections were stained with 0.1% cresyl violet solution for 5 to 10 min. Then, the sections were washed with 0.8% acetic anhydrate in 90% ethanol for 5 to 10 min to remove excess cresyl violet.

### Data Analysis

The slides were scanned with a VS-120 automated brightfield microscope (Olympus, Tokyo, Japan). Then, image editing software of Adobe Photoshop (cc 2018, Adobe, San Jose, CA) and ImageJ (1.48v/Java 1.6.0_20) was used to edit figures. We did not digitally add any staining pattern in the figures besides enhancing brightness/contrast and putting some annotations and nomenclature. Some of the figures were too light for the microscope to focus on the signals easily, we used USM tool in photoshop (cc 2018, Adobe, San Jose, CA) to enhance resolution. Those parameters were modified carefully to avoid distortion. In addition, the contour of blobs and stripes was revealed roughly by photoshop (cc 2018, Adobe, San Jose, CA) automatically first, then revising the contour closely and manually with the guidance of the rough lines.

**Figure 1:**
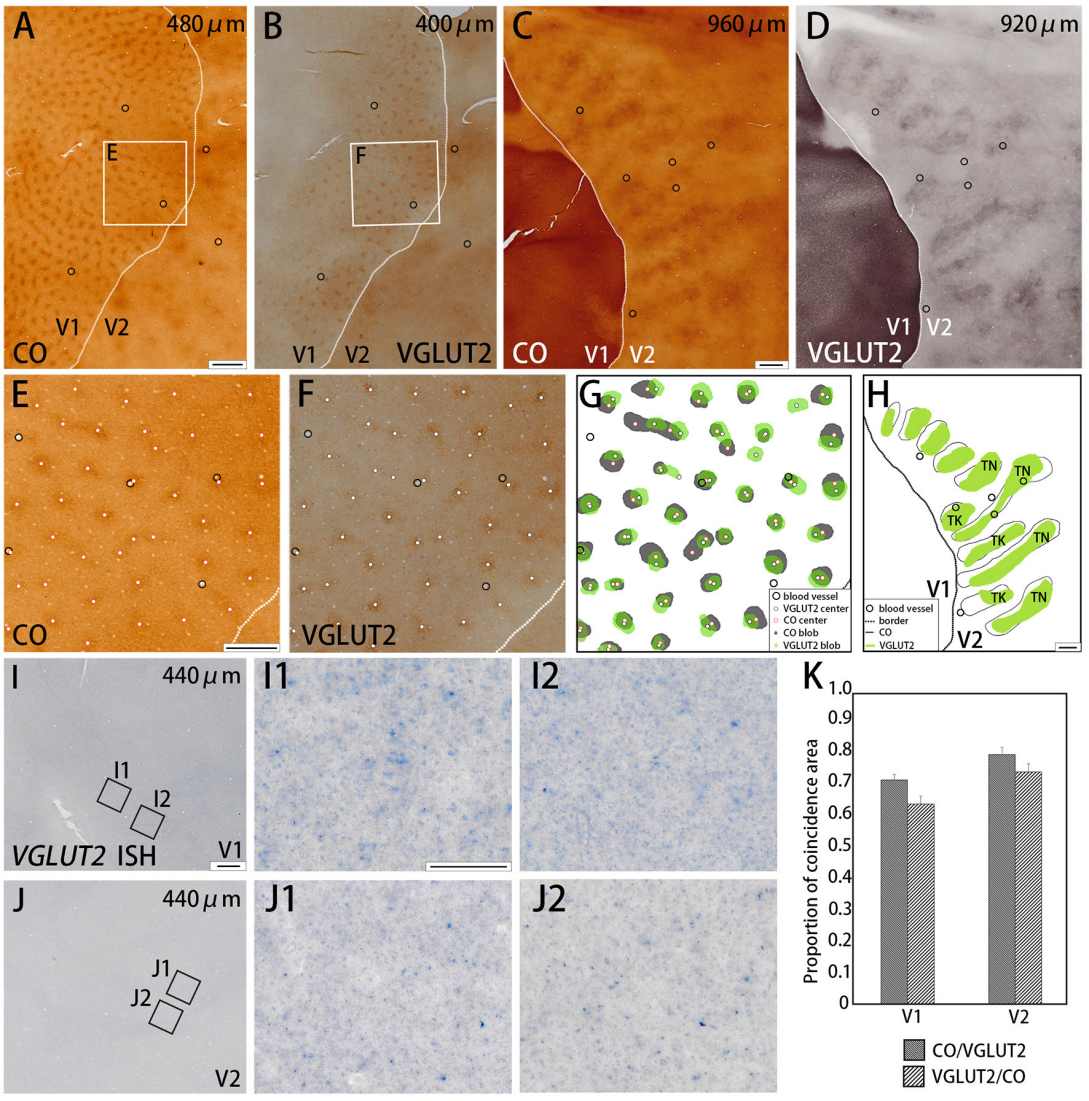
Tangential sections of V1 and V2 stained for CO activity **(A,C)** or VGLUT2-immunoreactivity (ir) **(B,D)** in the squirrel monkey (Case ID 18–27, right hemisphere). **(A,B)** are adjacent with each other in superficial layers, and **(C,D)** are adjacent with each other in middle layers. **(E,F)** Higher magnification of regions of interest (rectangle area) in **(A,B)**, respectively. Black circles in **(A–E)** indicate corresponding blood vessels of adjacent sections for alignment. White dots in **(E,F)** indicate centers of patchy patterns for each staining. **(G,H)** Comparison of V1 blob **(G)** or V2 stripe **(H)** patterns obtained from each staining. “TN” is for thin stripe, and “TK” is for thick stripe. Other annotations are as indicated in the inset. **(I,J)** A tangential section of V1 and V2 stained for mRNA of *VGLUT2* gene, respectively. **(I1–J2)** Higher magnifications of regions of interest in V1 **(I1,I2)** and V2 **(J1,J2)** (rectangle area in **I,J**). Only a few *VGLUT2* mRNA signals are seen. **(K)** Bar chart of the proportion of coincidence area in both V1 and V2. Dashed lines indicate borders between V1 and V2. Depth from the pial surface of each section is indicated at the upper-right corner in **(A–D,I,J)**. Scale bars are 1 mm for **(A–D,G,H)**, 500 μm for **(E,F)**, 500 μm for **(I,J)**, and 50 μm for **(I1–J2)**.

To count the coincidence area ratio, we chose 43 blobs and 29 different kind of stripes randomly in four hemispheres of three cases, which had been shown in Figures 1G,H, 4C,F,I,L. The area of CO blobs and VGLUT2-ir patches were calculated on Photoshop, the data was processed by Excel (Microsoft) and IBM SPASS Statistics (version 20) and the bar chart was drawn on Excel. CO/VGLUT2 was calculated by using the coincidence area between one single CO blob and VGLUT2-ir patch divided by the VGLUT2-ir patch area. VGLUT2/CO was calculated by using the coincidence area between one single CO blob and VGLUT2-ir patch divided by the CO blob area. The results represent the proportion of coincidence area (Figure 1K). To objectively evaluate overlap proportion between CO blobs and VGLUT2-ir patches in V1, and between CO dark stripes and VGLUT2-ir stripes in V2, the contours of staining patterns were drawn using MATLAB (R2020a 9.8.0.1323502). After normalization of the original figures, a series of functions were applied to optimize pictures, such as Gaussian blur, followed by the function of contour to show the outline of different patterns automatically, then overlap proportion was calculated (Figure 5).

**Figure 2:**
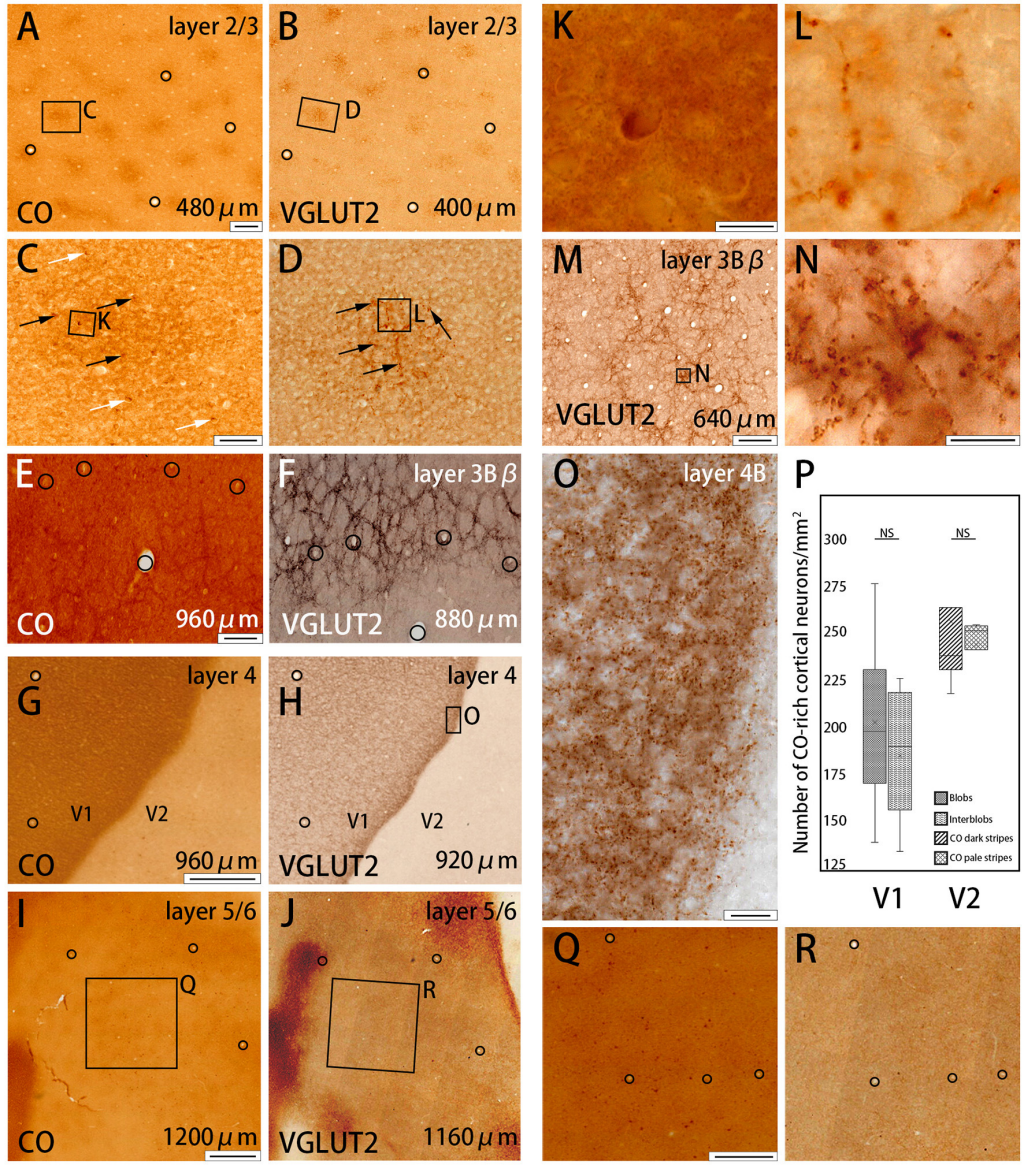
Higher magnifications of CO histochemistry **(A,E,G,I)** and VGLUT2 IHC **(B,F,H,J,M)** in tangential sections of V1 (Case ID 18–27, right hemisphere). **(A,B,E–J)** are adjacent to each other, and corresponding blood vessels are circled. Approximate layers and depth from the pial surface are indicated in each panel. **(C,D)** Even higher magnifications of the blob area that is boxed in **(A,B)**, respectively. CO-rich neurons are pointed by black or white arrows within blob or outside blob, respectively, in **(C)**. VGLUT2-ir positive neuropils and puncta in blob are pointed by black arrows in **(D)**. Corresponding vessel patterns in different layers were plotted between **(E,F)** [layer 3Bβ (4A)], **(G,H)** [layer 4 (4C)], and **(I,J)** (layer 5/6). In **(E,F)**, the location of the honeycomb structure appears mismatched between CO and VGLUT2-ir in the regard of vasculature patterns. That is because the flattening was not complete in this part of the large tangential section, and layer 3Bβ (4A) was thin, therefore, honeycomb structure was slightly displaced even when the distance of the two sections was only 80 μm apart. **(K,L)** Even higher magnification of a single CO-rich neuron and boutons in **(C,D)**, (boxed area). **(N)** Even higher magnification of the honeycomb area that is boxed in **(M)**. **(O)** Even higher magnification of layer 4β (4Cβ) that is boxed in **(H)**. **(Q,R)** Even higher magnifications of infragranular blob area that is boxed in **(I,J)**, respectively. **(P)** Box-plot about the number of CO-rich cortical neurons in both V1 and V2 per mm^2^. NS indicates not significant. Depth from the pial surface is indicated at the right bottom in **(A,B,E–J,M)**. The scale bars are 200 μm for **(A,B,E,F,M)**, 50 μm for **(C,D)**, 20 μm for **(O)**, 10 μm for **(K,L,N)**, 500 μm for **(G,H,Q,R)**, and 1 mm for **(I,J)**.

As for counting the number of CO-rich cortical neurons, gridlines that both the length and width were 1 mm were used on all sample areas which size were around 5 mm by 5 mm, to help us select samples of blobs in V1 randomly, totally 78 blobs in all four hemispheres were chosen. In each blob, counting the number of CO-rich cortical neurons within a 0.2 × 0.2 μm square box. While in interblob, we chose 2–4 sample areas in the same square box area and calculated the average values. In V2, we chose ~20 stripes each of CO dark and pale stripes, and in each stripe, we selected several different samples in different area, in total, there were about 170 samples of those stripes (Figure 2P). We used unpaired Student *t*-test to evaluate statistics, and considered that it is significant difference when *p*-value < 0.05.

## Results

Some inconsistency exists in the literature concerning the numbering of V1 layers (Balaram et al., 2014). In previous and current studies in New World monkeys, we used Hässler's scheme of the V1 layering (Hässler, 1967; Takahata et al., 2012), where only layer 4C of Brodmann is considered to be layer 4, and provided Brodmann's layers in parenthesis.

### Coincidence Between CO Histochemistry and VGLUT2-ir in V1

We examined the CO reactivity and immunoreactivity (ir) for VGLUT2 on the flattened visual cortex from squirrel monkeys. In the superficial layers (within 500 μm of the pial surface) of V1, a regular patchy pattern of CO-rich compartments was revealed with an interval of 0.3 to 0.5 mm and diameters that ranged from 0.1 to 0.3 mm (Figure 1A). This observation was consistent with the previously reported pattern of CO blobs (Carroll and Wong-Riley, 1984; Horton, 1984). VGLUT2-ir in adjacent sections exhibited a very similar pattern to the pattern of CO staining (Figure 1B). Using higher magnification, the centers of each blob were plotted for CO staining and VGLUT2-ir to examine their correspondence (Figures 1E,F). These adjacent images were digitally arranged according to the corresponding blood vessels in each section to compare the shape, size, and locations of the CO blobs and VGLUT2-ir patches (Figure 1G). We observed that the majority of the CO blobs and VGLUT2-ir patches were coincident to a considerable extent. To quantify our observation, we demarcated CO blobs and VGLUT2-ir patches, and calculated overlapping areas (Figure 1K). As a result, 72.5 ± 1.8% (mean ± S.E.M., *n* = 3, Does as follows) of CO blob area was coincided with VGLUT2-ir patches, and 64.8 ± 0.1% of VGLUT2-ir patch area was coincided with CO blob area (calculated for 43 CO blobs/VGLUT2-ir patches from four hemispheres of three monkeys), indicating that a majority of each area overlaps with each other. We then examined mRNA expression of the *VGLUT2* gene in the visual cortex to address whether cortical neurons were the origin of VGLUT2 protein. Overall, the mRNA signal was sparse, and blob-like pattern was not observed in V1 (Figure 1I). Some faint signal was visible at higher magnification, which indicated that the ISH staining worked properly (Figures 1I1,1I2). The mRNA expression pattern suggested that the VGLUT2 protein that composed the V1 blob pattern originated from brain regions other than the visual cortex.

To further study the microstructure in and surrounding the CO blobs, we obtained images of layers 2/3 of V1 with high-power magnification (Figures 2A–D). Somas of cortical neurons that were rich in CO activity were scarce (black and white arrows in Figures 2C,K), and there was no apparent difference in their distribution or population when the insides of the blobs were compared to the areas surrounding the blobs. To quantify this observation, we demarcated CO blob boundaries, and separately counted the number of CO-rich cells in and surrounding the CO blobs (Figure 2P). The number of CO-rich cells in CO blobs was 207 ± 29.5 per mm^2^ [counted for 253 regions of interest (ROIs) of 0.2 × 0.2 mm square from 78 CO blobs in 4 hemispheres of 3 monkeys], whereas that outside CO blob was 189.25 ± 22.3 per mm^2^ (counted for 318 ROIs of 0.2 × 0.2 mm square from 78 interblobs in 4 hemispheres of 3 monkeys), and there was no statistical significance between them (*p* = 0.64, *n* = 3). This result suggested that CO-rich cortical neurons did not constitute a major component of the CO blobs. CO intensity was higher overall in the blobs compared to the areas surrounding the blobs, but the detailed structure was unclear in the CO histochemistry (Figures 2C,K). The more precise microstructure of the blobs was apparent in VGLUT2 IHC compared to the CO histochemistry (Figures 2D,L). In VGLUT2 IHC, intermingled neuropil and puncta were visible in the blobs (black arrows in Figure 2D), which appeared to be axon terminals and boutons, respectively (Figure 2L). A collection of principal arbors and smaller secondary arbors formed a net-like structure. Few cortical cell somas were observed with VGLUT2 IHC. Taken together, these results suggested that the V1 CO blobs contain mainly VGLUT2 protein-containing axonal terminals and boutons that maintained higher metabolic activity than cortical neurons.

The presence of the characteristic honeycomb-like lattice structure revealed using CO histochemistry in layer 3Bβ (4A) has been confirmed in several previous studies (Carroll and Wong-Riley, 1984; Boyd et al., 2000). VGLUT2 IHC reportedly can reveal a honeycomb structure in macaques (Garcia-Marin et al., 2014). As expected, we observed a prominent honeycomb structure using both CO histochemistry and VGLUT2 IHC in squirrel monkeys (Figures 2E,F). Interestingly, VGLUT2 IHC demonstrated the pattern even more clearly than CO histochemistry (Figure 2M). Higher magnification illustrated that the honeycomb structure was produced by accumulations of dense neuropil and bouton-like puncta (Figure 2N). In both CO histochemistry and VGLUT2 IHC, the signals were most intense in layer 4 (4C) (Figures 2G,H). It was difficult to observe the microstructure due to high intensity, but similar to blobs, cell somas were rarely observed in VGLUT2 IHC. The signal was primarily located in neuropil and puncta in VGLUT2 IHC (Figure 2O). The CO labeling was nearly uniform throughout layer 4 (4C). CO blobs were mainly seen in the more superficial layers, but it has been reported that they have been observed in the infragranular layers in corresponding columns as superficial layers in squirrel monkeys (Carroll and Wong-Riley, 1984). The patchy CO expression was faint, but the similar pattern to the superficial blobs was observed in the infragranular layers of V1 in our samples (Figure 2I). VGLUT2-ir was also faintly patchy in the infragranular layers (Figure 2J). Their patterns and spacing appeared similar, although it was challenging to analyze the exact correspondence because the patterns were obscure (Figures 2Q,R). Similar results were obtained in the other hemisphere of the same animal and in the other squirrel monkeys (Figure 4).

### Coincidence Between CO Histochemistry and VGLUT2-ir in V2

Dark stripes (thick and thin stripes) and intercalated pale stripes were discernable throughout V2 in tangential CO stained sections (Figure 1C), as reported previously (Wong-Riley and Carroll, 1984b). Some dark stripes were slightly darker and thinner than others, which facilitated the ability to discriminate between thin and thick stripes (Figure 1H). The width of one thick-pale and thin-pale unit was ~2 mm. We observed that VGLUT2-ir revealed strikingly similar patterns as CO histochemistry (Figure 1D). To address whether the two staining methods were localized in the same V2 compartments, we used blood vessel patterns in each section to digitally align the stained images from adjacent sections, and observed the degree of overlap for the striped patterns (Figure 1H). A significant overlap was present between the stripes produced by CO histochemistry and VGLUT2 IHC, revealing that VGLUT2 IHC exhibited the same stripes that were revealed with CO histochemistry. To quantify our observation, we demarcated CO dark stripes and VGLUT2-ir stripes, and calculated overlapping areas (Figure 1K). 80.8 ± 1.4% of CO dark stripe area coincided with VGLUT2-ir stripes, and 75.1 ± 1.5% of VGLUT2-ir stripe area coincided with CO dark stripe area (calculated for 29 CO dark stripes/VGLUT2-ir stripes from four hemispheres of three monkeys), indicating that a majority of CO dark stripe overlaps with VGLUT2-ir rich stripe area. ISH for *VGLUT2* mRNA showed weak sparse signal, but it did not exhibit a stripe-like pattern (Figure 1J). This result indicated that the VGLUT2 protein that reveals the V2 stripe pattern had a different origin outside of V2.

**Figure 3:**
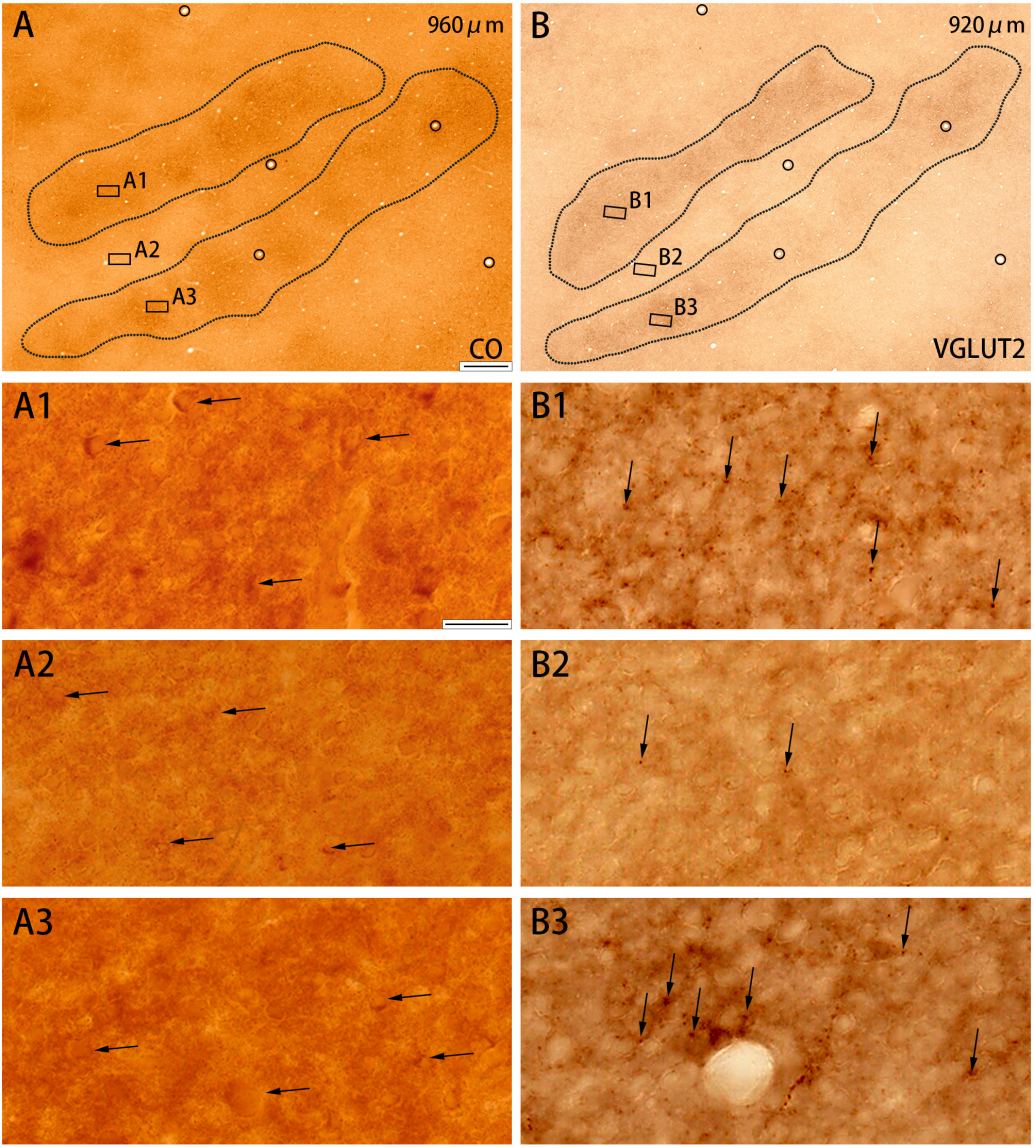
Higher magnifications of CO histochemistry **(A)** and VGLUT2 IHC **(B)** in tangential sections of V2 (Case ID 18–27, right hemisphere). **(A,B)** are adjacent to each other, and corresponding blood vessels are circled. Depth from the pial surface is indicated in each panel. Shapes of V2 stripes are delineated by black dotted lines. Three regions of each panel were chosen to enlarge as indicated **(A1–A3,B1–B3)**. CO-rich cortical cells are pointed by black arrows in **(A1–A3)** to illustrate that they are present in both CO dark **(A1,A3)** and pale **(A2)** zones. VGLUT2-ir positive neuropils and puncta are pointed by black arrows in **(B1–B3)** to illustrate that they are abundant in CO dark zones **(B1,B3)**, but scarce in CO pale zones **(B2)**. Scale bars are 500 μm for **(A,B)**, and 20 μm for **(A1–A3,B1–B3)**.

**Figure 4:**
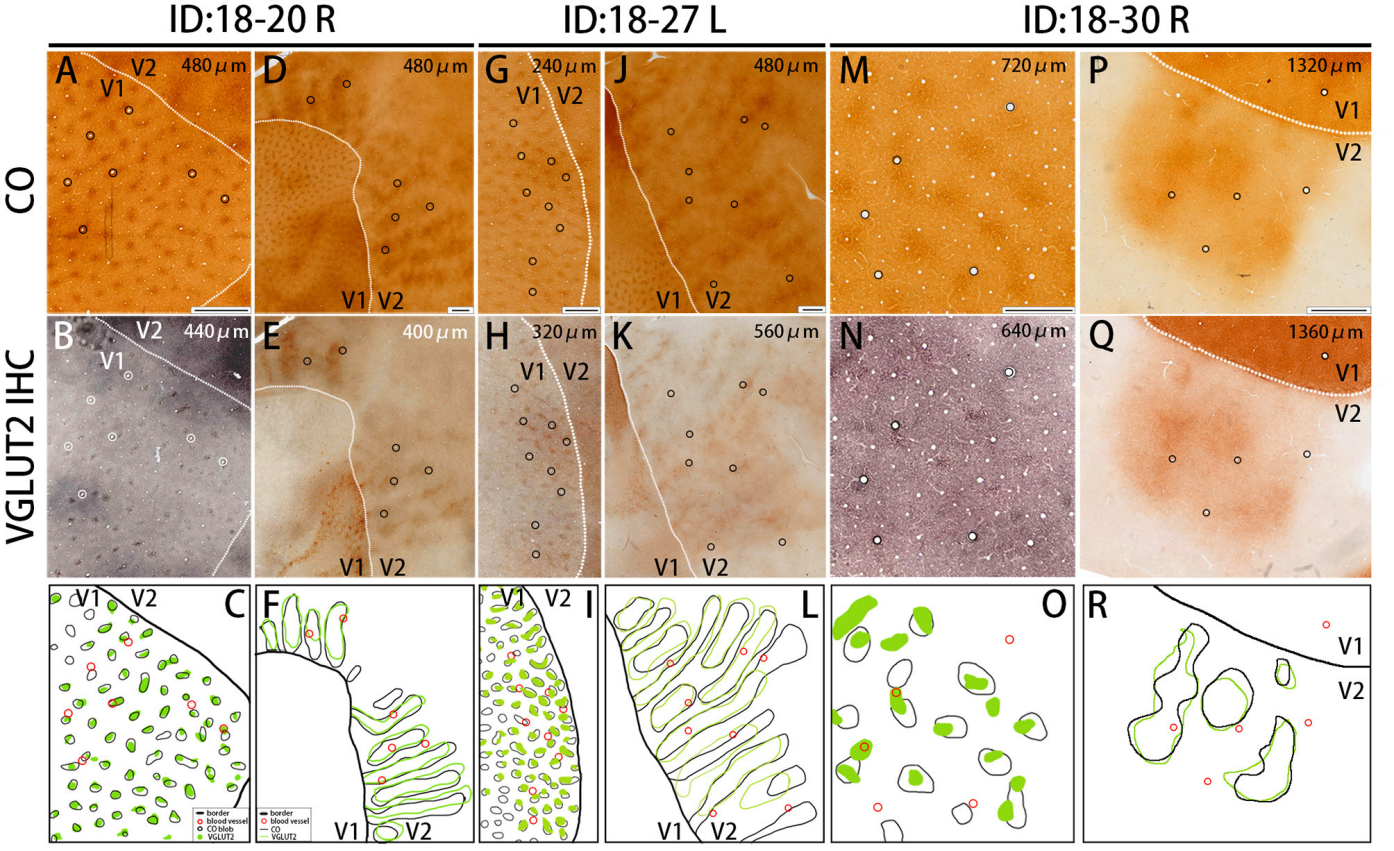
Similar results were obtained in other animals (Case ID 18–20, right hemisphere, **A–F**, and Case ID 18–30, right hemisphere, **M–R**), and in the other hemisphere of the same animal above (Case ID 18–27, left hemisphere, **G–L**). **(A,B,D,E,G,H,J,K,M,N,P,Q)** are adjacent to each other, and corresponding blood vessels are circled. Depth from the pial surface is indicated in each panel. White dotted lines indicate V1/V2 borders. Comparisons of V1 blob pattern between CO histochemistry and VGLUT2 IHC for ID 18–20 right, ID 18–27 left, and ID 18–30 right are shown in **(C,I,O)**, respectively, and comparisons of V2 stripe pattern between CO histochemistry and VGLUT2 IHC for ID 18–20 right, ID 18–27 left, and ID 18–30 right are shown in **(F,L,R)**, respectively. Annotations are as indicated in the inset. Scale bars are 500 μm for **(M–O)**, and 1 mm for others.

CO-rich cortical cells were observed in V2 at higher magnification (black arrows in Figures 3A1–A3). The CO-stained cortical cells were present in both CO dark (thick and thin) stripes and pale stripes. However, independent of the presence or absence of CO-rich cells, the CO dark stripes were darker overall than the CO pale stripes, and no particular microstructure was present. To quantify this observation, we demarcated CO stripe boundaries, and separately counted the number of CO-rich cells in and surrounding the CO dark stripes (Figure 2P). The number of CO-rich cells in CO dark stripes was 261.5 ± 25.5 per mm^2^ (counted for 92 ROIs of 0.2 × 0.2 mm square from 20 CO dark stripes in 4 hemispheres of 3 monkeys), whereas that outside CO dark stripes was 249 ± 9.0 per mm^2^ (counted for 82 ROIs of 0.2 × 0.2 mm square from 18 CO pale stripes in 4 hemispheres of 3 monkeys), and there was no statistical significance between them (*p* = 0.66, *n* = 3). This result implied that the presence of CO-rich cortical cells did not cause the pattern of thick and thin stripes. On the other hand, the VGLUT2-ir neuropil and puncta were abundantly observed in the thick and thin stripes but not in the pale stripes (Figures 3B1–B3) and VGLUT2-ir cortical cells were rare. These observations suggested that the thick and thin stripes in V2 contain mainly VGLUT2 protein-containing axonal terminals and boutons that were more metabolically active than cortical neurons. Similar results were obtained in the other hemisphere of the same animal and in the other squirrel monkeys (Figure 4).

**Figure 5:**
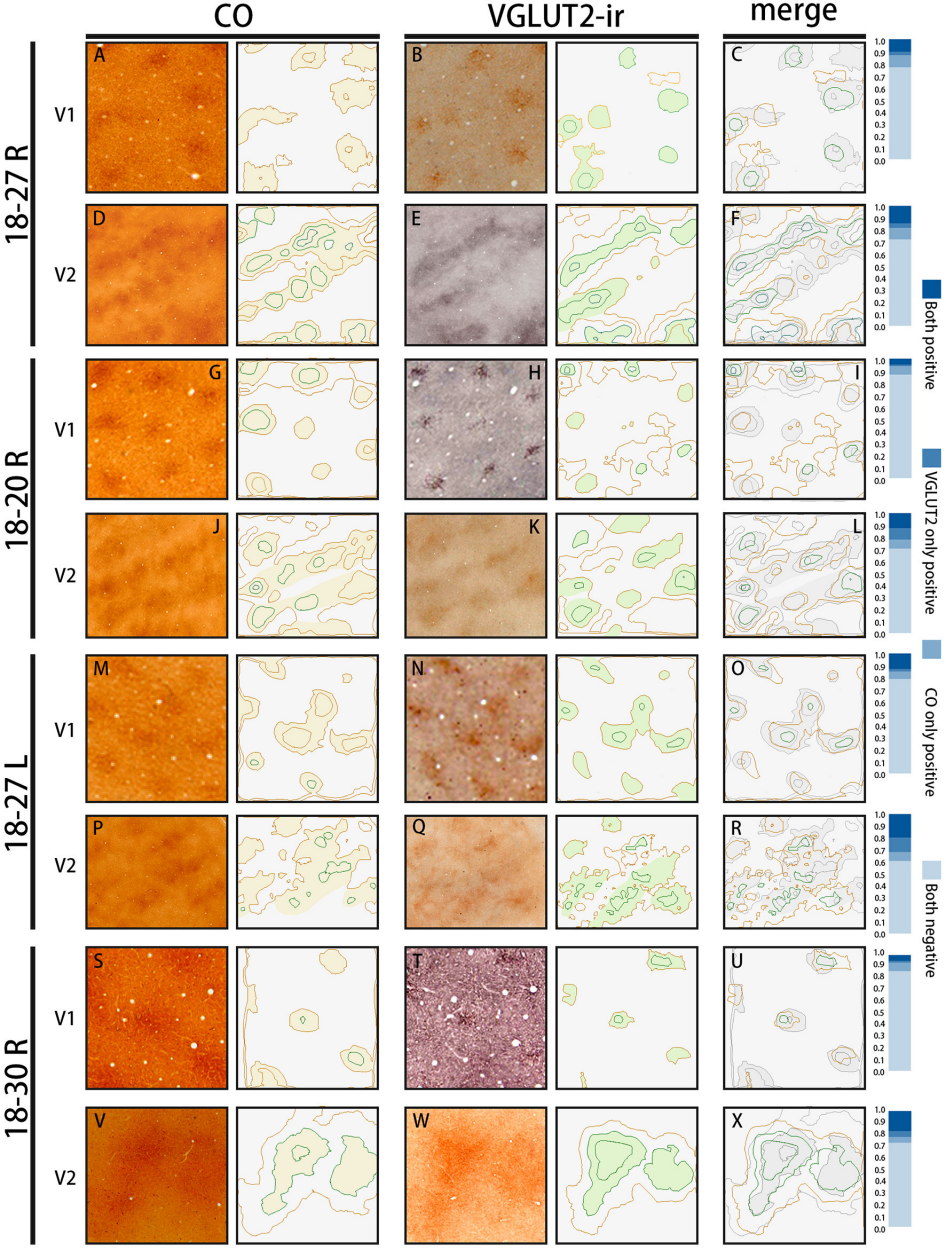
The automated evaluation of overlap proportion between CO blobs and VGLUT2-ir patches in V1, and between CO dark stripes and VGLUT2-ir rice stripes in V2 in all three hemispheres examined. The original images are on the left, and their contours drawn by the program are on the right. **(A,D,G,J,M,P,S,V)** are CO-stained sections, and **(B,E,H,K,N,Q,T,W)** are sections stained for VGLUT2-ir, which are adjacent to the sections of CO histochemistry on their left. They are closely aligned with each other to match vasculature patterns. The estimated CO or VGLUT2-ir dark areas are lightly colored. **(C,F,I,L,O,R,U,X)** Merged contours between the two staining on their left. The CO dark areas are colored with light gray for the ease of comparison. The bars on the right show proportions of the overlap between the two staining methods. “Both positive” indicates the proportion of areas that are rich in both CO and VGLUT2-ir, “VGLUT2 only positive” indicates the proportion of areas that are rich in VGLUT2-ir but not in CO, “CO only positive” indicates the proportion of areas that are rich in CO but not in VGLUT2-ir, and “Both negative” indicates the proportion of areas that are not rich in CO or VGLUT2-ir.

The quantification of overlap in V1 blobs and V2 stripes was done with manual drawing of contours. To confirm that the results were not biased by subjectivity of investigators, we used an automated program to quantify the proportion of overlap in selected small regions (Figure 5), whereas this analysis was difficult to be applied to large tangential sections due to the contamination of different layers, uneven staining intensity and distraction of staining patterns by vessels. In the right V1 of ID 18–20, the CO blobs/VGLUT2-ir patches was 0.83 and the VGLUT2-ir patches/CO blobs was 0.42. Average proportion of the CO blobs/VGLUT2-ir patches in the right and left V1 of ID 18–27 was 0.72 and that of the VGLUT2-ir patches/CO blobs was 0.59. In the right V1 of ID 18–30, the CO blobs/VGLUT2-ir patches was 0.71 and the VGLUT2-ir patches/CO blobs was 0.42. Altogether, the ratio of CO blobs/VGLUT2-ir patches was 75.5 ± 5.5% and that of VGLUT2-ir patches/CO blobs was 47.4 ± 8.2% (*n* = 3 each). In the right V2 of ID 18–20, the CO dark stripes/VGLUT2-ir rich stripes was 0.59 and the VGLUT2-ir rich stripes/CO dark stripes was 0.62. Average proportion of the CO dark stripes/VGLUT2 in the right and left V2 of ID 18–27 was 0.69 and the VGLUT2-ir rich stripes/CO dark stripes was 0.66. In the right V2 of ID 18–30, the CO dark stripes/VGLUT2-ir rich stripes was 0.77 and the VGLUT2-ir rich stripes/CO dark stripes was 0.77. Altogether, the ratio of CO dark stripes/VGLUT2-ir rich stripes was 68.3 ± 7.4% and that of VGLUT2-ir rich stripes/CO dark stripes was 68.4 ± 6.5% (*n* = 3 each). Overall, significant overlap was observed in this analysis as well.

### Thalamic Origin of VGLUT2 Protein

To address the possibility that the VGLUT2 protein observed in V1 and V2 was derived from the thalamus, we used ISH for *VGLUT2* mRNA to study the lateral geniculate nucleus (LGN) and the pulvinar complex. Even though we attempted monocular inactivation using TTX injection (1 mM−3 μL) into the left eye in this specific animal (ID 19-04) 2 days before perfusion, no apparent difference in gene expression or CO staining pattern was observed between the different layers or hemispheres. Therefore, we considered that the histochemical patterns in this subject were similar to those of visually intact animals.

**Figure 6:**
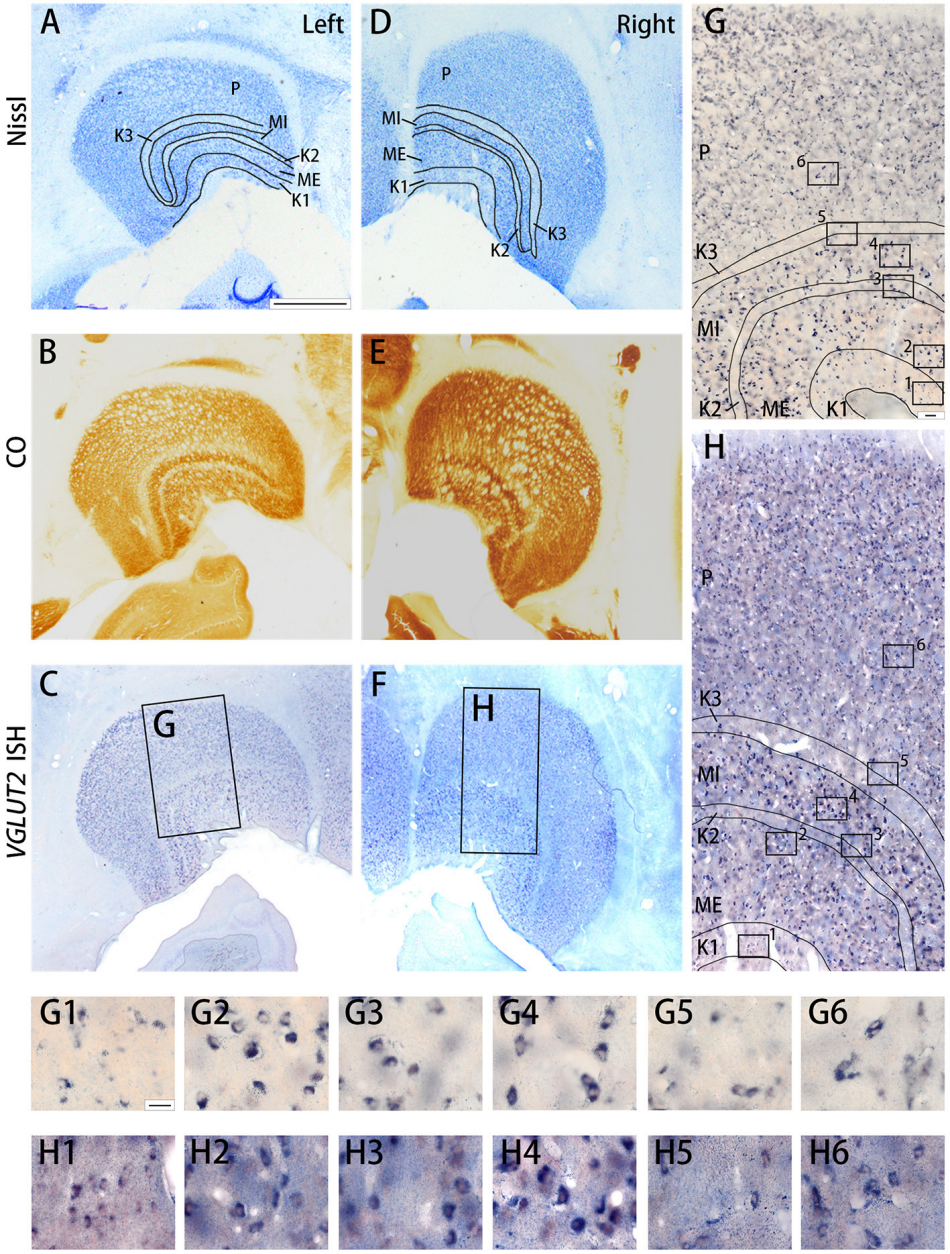
Coronal sections of the LGN (Case ID 19–04) stained for Nissl substance **(A,D)**, CO **(B,E)**, *VGLUT2* mRNA **(C,F)**. **(A–C)** is in the left hemisphere and left is lateral, and **(D–F)** is in the right hemisphere and left is medial. The sublayers of the LGN were identified according to Nissl staining and CO histochemistry, including K1, K2, K3, MI, ME, and P, which are illustrated by black lines. **(G,H)** High magnification of the rectangle areas in **(C,F)**, respectively. Even higher magnification in **(G,H)** are shown as **(G1–G6,H1–H6)** below. Scale bars are 1 mm in **(A–F)**, 50 μm for **(G,H)** and 20 μm for **(G1–G6,H1–H6)**.

The squirrel monkey LGN consists of magnocellular (M) layers, parvocellular (P) layers, and koniocellular (K) layers, as seen in other primates (Hess and Edwards, 1987; Usrey and Reid, 2000) (Figure 6). However, unlike other primate species, in the squirrel monkey, the P layers do not exhibit clear septa or intercalated layers, which causes the entire P layer to appear uniform. On the other hand, the cell morphology and organization of the M layers are similar to those observed in macaques (Schiller and Malpeli, 1978). The ventral M layer (ME) predominantly receives inputs from the contralateral eye, and the dorsal M layer (MI) predominantly receives inputs from the ipsilateral eye (Horton and Hocking, 1996; Usrey and Reid, 2000). A previous study reported that the K layers were seen ventral to M layers (K1), between two M layers (K2), and between P and M layers (K3) (Ding and Casagrande, 1997). Among them, the cells were quite sparse in K2 and K3, whereas more cells were observed in K1 (Figures 6A,D). CO was active in all the LGN layers (Figures 6B,E) Robust *VGLUT2* mRNA signal was observed in all LGN layers (Figures 6C,F). Based on the cell morphology observed at high magnification, it was determined that the mRNA signals mainly resided in dense, large excitatory relay neurons of the LGN (Figures 6G,H).

**Figure 7:**
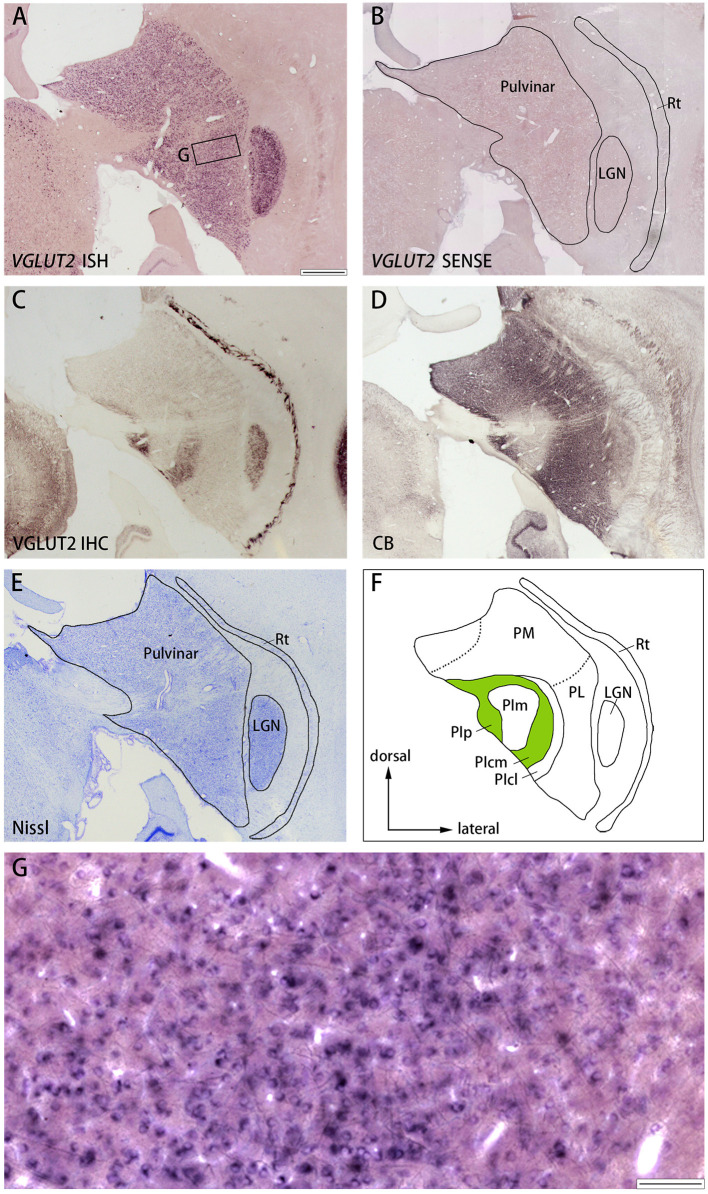
**(A–E)** Coronal sections of the pulvinar (Case ID 18–31, right hemisphere) stained for *VGLUT2* mRNA **(A)**, *VGLUT2* sense probe **(B)**, VGLUT2-ir **(C)**, calbindin D-28K (CB)-ir **(D)**, or Nissl substance **(E)**. Left is medial and upper is dorsal. **(F)** Nuclei of the pulvinar complex were identified according to Nissl staining, VGLUT2 IHC and CB IHC, following Baldwin et al. (2017). **(G)** High magnification of the rectangle area in **(A)**. Scale bars are 1 mm in **(A–F)** and 100 μm for **(G)**. PM, medial pulvinar; PL, lateral pulvinar; PIp, posterior inferior pulvinar; PIm, middle inferior pulvinar; PIcm, central medial inferior pulvinar; PIcl, central lateral inferior pulvinar; Rt, thalamic reticular nucleus; LGN, lateral geniculate nucleus.

Similar results were observed in the pulvinar. In Figure 6, the results in ID 18–31 (female, 800 g), which was subjected to monocular inactivation treatment by left eye enucleation for 20 days before perfusion, are presented. The pulvinar nuclei, medial pulvinar (PM), lateral pulvinar (PL), and inferior pulvinar (PI) were identified using IHC for calbindin D-28K (CB) and VGLUT2 as well as Nissl staining (Figures 7C–F), as described previously (Baldwin et al., 2017). The *VGLUT2* mRNA signals were abundantly and uniformly observed in all pulvinar nuclei (Figure 7A). Neuronal expression was confirmed using high magnification (Figure 7G). In general, VGLUT2-ir was relatively low, except in the posterior PI (PIp) and caudal medial PI (PIcm), which receive direct projections from the superior colliculus (SC) (Baldwin et al., 2013) (Figure 7C). The *VGLUT2* sense probe did not reveal any signal above background (Figure 7B).

## Discussion

The purpose of this study is to demonstrate how the expression patterns of CO and VGLUT2-ir correspond with each other. The close correspondence between labeling patterns of the two staining methods supported the possibility that CO-rich domains such as V1 blobs and V2 thick and thin stripes in the primate visual cortex are distinct because they contain abundant afferent terminals from the thalamus. VGLUT2 is an excellent endogenous molecule to study thalamo-cortical connectivity patterns because it is strongly expressed in primary neurons of all sensory thalamic nuclei, but not in the cortex. Also, a majority of the VGLUT2 protein is transported and stored in axon terminals (Nahmani and Erisir, 2005; Nakamura et al., 2007; Balaram et al., 2013; Garcia-Marin et al., 2013; Hackett et al., 2016). Parvalbumin has also been used to study thalamo-cortical afferent distribution (Spatz et al., 1994; Melchitzky et al., 1999; Wong and Kaas, 2010), but it is not the best molecule to use because it is also strongly expressed in cortical GABAergic interneurons (DeFelipe et al., 1999). There is a concern about influence of monocular inactivation treatment undergone in our subjects on the expression of CO and VGLUT2-ir. There are studies that show a dramatic decrease of their expressions following monocular inactivation in the primate visual system (Wong-Riley and Carroll, 1984a; Horton and Hocking, 1998a; Takahata et al., 2018), and some structural changes also occur in CO blobs and ocular dominance columns even in adult cases (Trusk et al., 1990; Rosa et al., 1991; Farias et al., 2019). Thus, their expression patterns may be slightly different from those in naïve animals, however, a remarkable correspondence between CO histochemistry and VGLUT2-ir in the visual cortex was shown, and we consider that our original purpose has been achieved whatever the condition of the animal was.

### Parallel Visual Pathways of the Geniculo-Striate Projection

Visual information of different modalities is processed separately in parallel pathways of the LGN and visual cortex (Lund, 1988; Hendry and Reid, 2000; Callaway, 2005; Sincich and Horton, 2005; Lu and Roe, 2008), as described in the introduction. Details of object shapes with high spatial frequency are mainly processed in the parvocellular layers of the LGN and relayed into layers 3Bβ (4A) and 4b (4Cβ) of V1, while object movements with high temporal frequency are mainly processed in the magnocellular layers of the LGN and relayed into layer 4α (4Cα) of V1. Color information, especially from the S cones of the retina, is processed in the koniocellular layers of the LGN and relayed into blobs of V1 (Hendry and Reid, 2000). Previous studies have shown that the distinct honeycomb structure observed as a repetition of circular neuropil and cell-sparse centers in layer 3Bβ (4A) of V1 corresponds with axonal terminals derived from koniocellular and parvocellular layers of the LGN (Hendrickson et al., 1978; Ding and Casagrande, 1998). *VGLUT2* mRNA is robustly expressed in all LGN layers, and VGLUT2-ir is distinctly observed in all V1 layers and domains described above. Although previous studies suggested that there is some projection from the pulvinar to layer 1 of V1 (Moore et al., 2018), V1 does not receive other major subcortical inputs, except minor inputs from the amygdala, claustrum, and nucleus basalis of Meynert (Hendrickson et al., 1978; Kennedy and Bullier, 1985). On the other hand, the LGN does not send major afferents to regions other than V1, whereas it has been shown that the koniocellular layers send minor projections into extrastriate visual cortices and MT (Lyon et al., 2010; Baldwin et al., 2012). Thus, we considered that the intense VGLUT2-ir and CO activity observed in V1 mainly represented major inputs from the LGN.

### Pulvino-Cortical Projection and Extrastriate Visual Cortices

Traditional tracer studies have revealed that feedforward projections from the pulvinar complex terminate in the middle layers of CO-rich thick and thin stripes in V2 (Livingstone and Hubel, 1982; Levitt et al., 1995). A similarity between CO and VGLUT2-ir patterns in V2 has also been described previously in the regard of possible pulvino-extrastriate terminals into layer 4 (Balaram et al., 2013, 2014). Thick stripes are considered to be a part of the M pathway and receive inputs from the interblob of V1, whereas thin stripes are considered to be a part of the P pathway and receive inputs from the blobs of V1 (Sincich and Horton, 2005). On the other hand, it has also been suggested that interstripes receive their major afferent inputs from V1, whereas thick and thin stripes receive their major afferent inputs from the pulvinar (Sincich and Horton, 2002b). Some studies have revealed that V2 receives some inputs arising from the LGN koniocellular layers (Hendrickson et al., 1978). V2 does not receive any other major subcortical afferent inputs, except minor inputs from the amygdala, claustrum, and nucleus basalis of Meynert as V1 (Kennedy and Bullier, 1985; Ungerleider et al., 2014). The VGLUT2-immunoreactive microarchitecture observed in this study resembled the appearance of pulvino-cortical terminals that were shown in a previous study where they carefully tracked and illustrated branches and terminals of a single axon arising from the PL (Rockland et al., 1999). Thus, we concluded that the CO staining density and VGLUT2-ir in V2 thick and thin stripes were mainly derived from the pulvinar.

Furthermore, we consider that this principle could be applied to more extrastriate visual cortices beyond V2. The pulvinar complex of primates consists of multiple nuclei, and the PL primarily sends axons into V2 (Ungerleider et al., 2014; Baldwin et al., 2017). The PL also sends afferent axons in other extrastriate visual cortices (Lyon et al., 2010; Gattass et al., 2014). The MT is the main target for the PI, and the PM is thought to innervate, more broadly, the posterior parietal cortex and other sensory cortices (Baldwin et al., 2017; Mundinano et al., 2018). Therefore, V2 is not the sole target for the pulvinar. All extrastriate visual cortices including the posterior parietal and temporal cortex show a slightly higher CO density in deeper layer 3 and layer 4 compared to other layers, and the CO density gradually decreases along the ventral visual pathway (Tootell et al., 1985; Paxinos et al., 2009). The MT is reported to possess a slightly higher CO staining intensity, especially in deeper layer 3 and layer 4 (Tootell et al., 1985; Kaskan and Kaas, 2007). Although we did not include these areas in the present study, it is likely that the higher CO density in the middle layers of these cortical areas resides in afferent terminals that arise from the pulvinar.

Finally, the staining patterns were quite different between ISH and IHC for VGLUT2 in the pulvinar (Figures 7A,C). The localization of the protein is different from that of mRNA likely because the VGLUT2 protein that is produced in the pulvinar neurons is transported from the pulvinar into axon terminals in the cortex, while the VGLUT2 protein that is produced in the SC neurons is transported into axon terminals in the PIp and PIcm. Moreover, CO histochemistry and VGLUT2 IHC produces complimentary results in the pulvinar: PIm is stained strongly for CO, distinguishing it from PIp and PIcm, both of which are stained relatively weakly for CO (Balaram et al., 2013), whereas VGLUT2-ir is robust in PIp and PIcm (Figure 7C) (Baldwin et al., 2017), which appears discrepancy to our current theory. This is reasonable, however, because the correspondence of staining patterns between CO and VGLUT2 IHC is only observed in the cortex due to the presumable imbalance of metabolism between the thalamus and the cortex as discussed below. Therefore, CO and VGLUT2-ir patterns do not correspond with each other in subcortical nuclei.

### Interpretation of CO Histochemistry

We consider that thalamic neurons generally possess higher metabolic activity than cortical neurons. Therefore, when the cortex is stained using CO histochemistry, clusters of the thalamo-cortical projection terminals may stand out compared to other regions (Takahata, 2016). Nonetheless, we do not deny the previous understanding that CO activity is coupled with neuronal spiking activity (Wong-Riley, 1989). CO activity and neuronal spiking activity are coupled, and CO histochemistry is an excellent method to study neuronal activity change, such as revealing ocular dominance columns after monocular loss (Deyoe et al., 1995; Horton and Hocking, 1998b). Notwithstanding, we consider that CO histochemistry reveals decreased activity in thalamo-cortical axons, or decreased activity in dendrites that receive direct projection from the LGN, after monocular inactivation, but not decreased activity in cortical neurons. CO activity might also change depending on the neuronal activity of cortical neurons, but any change is probably smaller than the difference in CO activity between thalamic and cortical neurons, and, therefore, is not clearly visible in the CO histochemistry. In other words, CO histochemistry reflects the activity of thalamo-cortical axons, but not the activity of cortico-cortical projection or local circuit activity.

Several electron microscopic studies suggested that CO-reactive mitochondria mainly reside in dendrites rather than axons in squirrel monkeys and macaques (Carroll and Wong-Riley, 1984; Wong-Riley and Carroll, 1984b; Wong-Riley et al., 1989), which argues against our theory. However, these studies were limited to layer 2/3 blobs of V1 or puffs (stripes) in V2, and did not examine the most CO-dense compartment of layer 4 (4C) of V1. The honeycomb structure in layer 3Bβ (4A) of V1 is prominently revealed using CO histochemistry, and the staining is likely due to geniculo-cortical axon terminals as described above. Besides, they did not distinguish thalamo-cortical synapses from cortico-cortical synapses, nor did they address the possibility that CO histochemistry might or might not represent major thalamo-cortical inputs. Another possible interpretation is that CO activity might reside in dendrites throughout the visual cortex as they suggested, but the CO histochemistry might represent CO activity in dendrites that receive direct inputs from the thalamo-cortical afferents, and this activity does not propagate into the soma. If that were the case, then the staining pattern would be the same as the distribution of thalamo-cortical afferent terminals. We have already discussed this possibility in our previous article (Takahata, 2016).

The similarity between CO staining pattern and thalamo-cortical afferent distribution has been pointed out in some previous studies as well (Livingstone and Hubel, 1982; Levitt et al., 1995; Sincich and Horton, 2002a). They discussed that thalamo-cortical afferents may activate cortical neurons strongly, contributing to higher expression of CO in cortical neurons. However, we slightly shift this idea to consider that dense CO activity resides in the thalamo-cortical afferents themselves, or in the cortical neuron dendrites that directly receive thalamic projection. This shift of interpretation is not trivial. For example, it suggests that neurons in V1 blobs and V2 thick and thin stripes are not necessarily more active than those in interblob or interstripe. Based on the idea that neurons in V1 blobs are more active than those in interblob, researchers previously presumed that neurons in V1 blobs lack orientation selectivity and respond to any orientation (Livingstone and Hubel, 1984; Edwards et al., 1995). However, our theory suggests that this is not true, which is consistent with many of the more recent studies (Lu and Roe, 2008; Economides et al., 2011; Garg et al., 2019). As another example of the importance of our theory, CO histochemistry only shows ODCs in layer 4 (4C) and shrinkage of layer 2/3 CO blobs after monocular blockade (Wong-Riley and Carroll, 1984a; Horton and Hocking, 1998b), suggesting that ocular dominance segregation is restricted to these regions. However, ocular dominance domains exist more broadly in other layers and even in extrastriate visual cortex in some species, as we have previously shown using activity-dependent gene expression (Takahata et al., 2009, 2014). As seen above, if one considers that CO histochemistry reveals spiking activity of cortical neurons, it can mislead conclusion of data, nonetheless, CO histochemistry is convenient staining to reveal distribution of thalamo-cortical afferent terminals and its activity.

## Data Availability Statement

The raw data supporting the conclusions of this article will be made available by the authors, without undue reservation.

## Ethics Statement

The animal study was reviewed and approved by Institutional Animal Care and Use Committee of Zhejiang University.

## Author Contributions

SY and TT designed research and wrote the manuscript. SY, QZ, SL, and TT conducted experiments. All authors contributed to the article and approved the submitted version.

## Conflict of Interest

The authors declare that the research was conducted in the absence of any commercial or financial relationships that could be construed as a potential conflict of interest.
